# The efficacy and safety of moxibustion for primary dysmenorrhea: A systematic review protocol

**DOI:** 10.1097/MD.0000000000018133

**Published:** 2019-11-27

**Authors:** Hao Wang, Xin Hui, Lue Ha, Baixiao Zhao, Qin Yao

**Affiliations:** Beijing University of Chinese Medicine, Beijing, China.

**Keywords:** meta-analysis, moxibustion, primary dysmenorrhea, protocol, systematic review

## Abstract

**Background:**

Primary dysmenorrhea (PDM) is one of the most prevalent gynecological diseases in women of childbearing age. The major medications treating PDM usually make sense and side effects, while moxibustion is known as a safe and effective treatment for PDM. This review aims to systematically evaluate the effect and safety of moxibustion for treating PDM.

**Methods:**

We will search all randomized controlled trials for moxibustion therapy on PDM, electronically and manually, regardless of publication status, till October 31, 2019. Online databases include the Cochrane Central Register of Controlled Trials; PubMed; EMBASE; China National Knowledge Infrastructure; Chinese Biomedical Literature Database; Chinese Scientific Journal Database (VIP database); and Wan-Fang Database. Two reviewers will search these databases, select data and measure the quality of studies independently. The methodological quality will be assessed by the Cochrane Reviewer's Handbook 6.0. The primary outcomes include clinical efficacy and visual analog scale, and the secondary outcomes include adverse events and quality of life. Four reviewers will independently extract the data and assess the qualities of the studies. Statistical analysis will be conducted with R package for each outcome. Study selection, data extraction, and assessment of risk of bias will be performed independently by 2 reviewers.

**Results:**

This study will provide a comprehensive review of the available evidence for the treatment of moxibustion with PDM.

**Conclusion:**

The conclusion of our study will provide updated evidence to judge whether moxibustion is an effective and safe intervention for patients with PDM.

**PROSPERO registration number:**

CRD42019129993

## 1 Introduction

Primary dysmenorrhea (PDM) is one of the most prevalent gynecological diseases in women of childbearing age. It refers to menstrual pain, often spasmodic, concentrated in the lower abdomen.^[1]^ Other symptoms include headache, dizziness, nausea and vomiting, diarrhea, and low back pain.^[2–4]^ It is a very common condition in young women. PDM is not associated with obvious pelvic organic disease and has high socioeconomic impact.^[5–10]^

So far, the cause of PDM has not yet been elucidated. Among the guideline^[11]^ issued by the Society of Obstetricians and Gynaecologists of Canada, the drugs currently used to treat PDM are acetaminophen, nonsteroidal anti-inflammatory drugs or oral contraceptives and complementary replacement therapies. However, nonsteroidal anti-inflammatory drugs or oral contraceptives may cause adverse effects on the liver, digestive tract, and other metabolic organs.^[12,13]^ Due to these shortcomings, moxibustion is a viable supplemental replacement therapy in China, Japan, the United States, and other regions. Years of application experience and widely used, especially in China, ongoing clinical trials have made moxibustion more and more accepted.^[14]^

Many researchers have conducted experiments on the mechanisms of moxibustion in the treatment of PDM. Ma et al^[15]^ analyzed herb-partitioned moxibustion could regulate the endocrine hormone to upregulated pregnenolone, prostaglandin E_2_, and γ-aminobutyric acid and downregulated the content of estrone and prostaglandin H_2_. Chen et al^[16]^ found after moxibustion treatment, uterine Prostaglandin F2α (PGF2α) content were obviously down and increase Natural Killer (NK) cell activity by regulating neuroendocrine-immune network. In the past, there are only 2 meta-analyses about moxibustion in the treatment of PDM, but their research had been 3 years and 5 years, respectively, and their focus is on the time of moxibustion intervention^[17]^ and all acupoint therapies including moxibustion.^[18]^ However, there is still no critically designed systematic review to assess the effectiveness and safety of moxibustion for PDM. Therefore, we will perform a systematic review of moxibustion for PDM to collect some reliable evidence for clinical guidance and to assistant PDM patients to seek more reasonable treatments. In this review, we aim to conduct a systematic review to evaluate all the clinical studies on the effects and safety of moxibustion for treating PDM in adults.

## 2 Methods

### 2.1 Study registration

This study has been registered at PROSPERO (registration number: CRD42019129993; http://www.crd.york.ac.uk/PROSPERO). This protocol is reported in compliance with the preferred reporting items for systematic reviews and meta-analysis protocols (PRISMA-P) statement guidelines.^[19]^ The review will be conducted in accordance with the PRISMA guidelines and follows the recommendations of the Cochrane Handbook for Systematic Reviews of Interventions.^[20,21]^ If we refine the procedures described in this protocol, we will update the record in the PROSPERO and disclose them in future publications related to this study.

### 2.2 Inclusion criteria for study selection

#### 2.2.1 Types of study

To evaluate the pure effect of moxibustion in the treatment of PDM, we will include all randomized controlled trials (RCTs) which explore the specific efficacy and safety of moxibustion in the treatment of PDM. Cross-trials, quasi-RCT, animal experiments, and other studies that were repeatedly published or did not have access to complete data will be excluded.

Trials will be excluded as follows:

(1) studies that involved a combination of moxibustion with other treatments, such as acupuncture, massage, cupping, and drugs or Chinese herbs;
(2) comparing moxibustion with other forms of acupuncture or other treatment;
(3) quasi-randomized trials and case reports.

#### 2.2.2 Types of participants

The included studies must be RCTs with patients diagnosed with PDM. PDM is defined as a lower abdomen pain that occurs during menses. Patients that were diagnosed with pelvic pathology or secondary dysmenorrhea will be excluded.

#### 2.2.3 Types of intervention

Any type of moxibustion therapy such as direct moxibustion, indirect moxibustion, heat-sensitive moxibustion, natural moxibustion, moxa-burner moxibustion, crude drug moxibustion as the sole treatment or a part of combination therapy with other intervention will be included. Studies in which moxibustion is not used as a major intervention will be excluded.

#### 2.2.4 Types of outcome measures

The primary endpoint evaluated will be the level of pain, with measurements made in the individual studies using the visual analog scale, numerical visual scale, or any other scale used for pain assessment. All response rates will be calculated from the total number of patients randomized. When the studies report response rates at various points of time, we decided a priori to compare the results obtained at the baseline with the endpoint of the treatment.

The secondary outcomes are adverse events and quality of life of women complaining of dysmenorrhea, who underwent moxibustion treatments. Symptoms often co-occurring with PDM include diarrhea, skin rash, and nausea. Diarrhea refers to abnormal bowel movements. Skin rash refers to some changes in the skin traits. Nausea refers to an urge to vomit. Quality of life was measured using questionnaires before and after treatment. When the studies report response rates at various points of time, we decided a priori to compare the results obtained at the baseline with the endpoint of the treatment.

### 2.3 Data sources

Our systematic review will search all RCTs for moxibustion therapy on PDM, electronically and manually, regardless of publication status, till October 31, 2019. Online databases include the Cochrane Central Register of Controlled Trials; PubMed; EMBASE; China National Knowledge Infrastructure; Chinese Biomedical Literature Database; Chinese Scientific Journal Database (VIP database); and Wan-Fang Database. Ongoing trials with unpublished data will be retrieved from the 4 following clinical trial registries: International Clinical Trials Registry Platform, NIH clinical registry Clinical Trials.gov, the Chinese clinical registry, and the Australian New Zealand Clinical Trials Registry. The reference lists of the selected studies and published systematic reviews will be screened for additional studies. Manually search for the grey literature, including conference proceedings.

### 2.4 Search strategy

A search strategy will be created according to the Cochrane handbook guidelines,^[20]^ and will be applied to all the electronic databases (equivalent search terms will be used in the Chinese databases).

The key search terms are (“primary dysmenorrhea” OR “algomenorrhea” OR “menalgia”) AND (“moxibustion” OR “moxa” OR “mox∗” OR “mugwort”) AND (“randomized”). The search strategy will be adapted to different databases demands. Search strategy in PubMed is shown in Table 1.

**Table 1:**
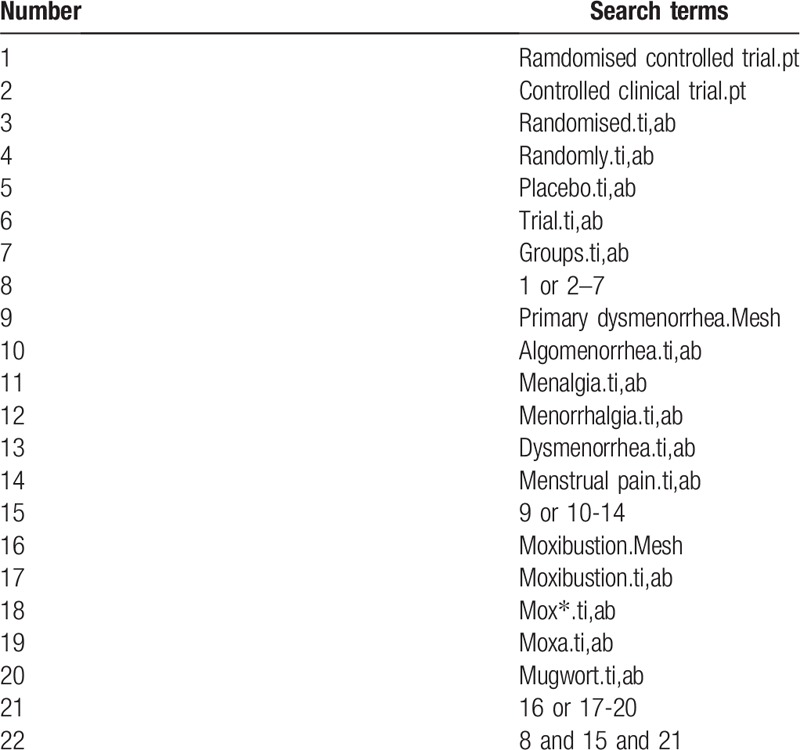
Search strategy used in PubMed.

### 2.5 Data collection and analysis

#### 2.5.1 Selection of studies

**Figure 1:**
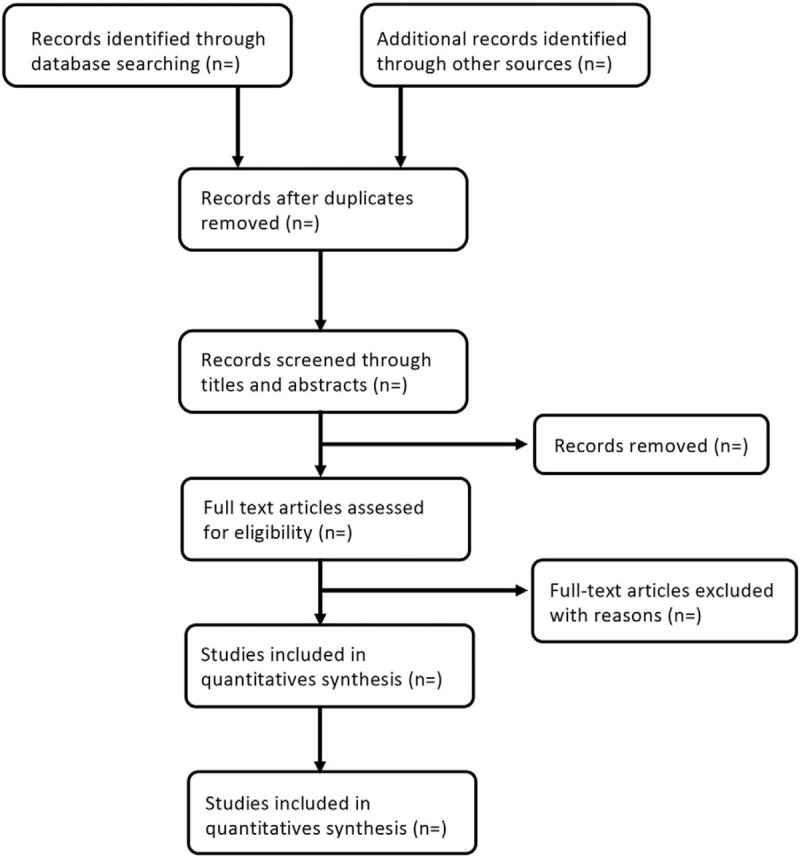
Flow diagram of the study selection process.

In the literature screening process, search results will be imported from the databases to EndNote V.X9, and this software will be used to remove duplicates and manage the trials that have been searched. Two reviewers (HW and XH) will independently review and screen the titles and abstracts of all retrieved studies to confirm eligible trials. The full text will be scanned if the studies cannot be identified after the screening of titles and abstracts. Excluded studies will be recorded with the reasons of their exclusion. In this process, disagreements will be discussed by the 2 authors (HW and XH) and be arbitrated by the third author (QY) when the 2 authors cannot reach consensus. The authors of the original studies will also be contacted for clarification when necessary. The process of the selection is shown in Figure 1.

#### 2.5.2 Data extraction and management

Before beginning extraction, a consistency assessment will be performed through a pilot test, in which each of them will evaluate 2 trials, respectively. After making a common consensus, 2 reviewers (QY and HW) will independently extract the data and assess the qualities of the studies. Data of the included studies will be collected, including first author, language used, study design, sample sizes, diagnostic criteria, patients’ ages, duration of disease, interventions used in the experimental group and control group, treatment duration, outcomes, adverse events, and type of moxibustion. Any discrepancy noticed in the process of data extraction will be resolved through discussion and the suggestion of a third reviewer (LH). For publications with insufficient or ambiguous data, we will attempt to obtain information from the corresponding authors by e-mail or telephone.

#### 2.5.3 Assessment of risk of bias and reporting of study quality

The Cochrane risk of bias tool, which is recommended by the Cochrane Reviewer's Handbook 6.0,^[20,21]^ will be used to evaluate the quality of the included studies.

HW and XH will independently evaluate the quality of selected articles from the following 5 aspects: selection bias (random sequence generation or allocation concealment), performance bias and detection bias (blinding), attrition bias (incomplete outcome data), reporting bias (selective outcome reporting), and other biases. If necessary, we will contact the corresponding author to clarify issues. The result of the consistency evaluation will be presented with Kappa statistics, Kappa value <0.75 will be considered the consistency have reached. Any disagreements will be resolved through discussion or consultation with BXZ.

#### 2.5.4 Data synthesis

Statistical analysis will be conducted with R package for each outcome. Relative risk, including 95% confidence intervals, will be used for the analysis of dichotomous data on clinical efficacy. If the *I*^2^ test is less than 50%, the fixed-effects model will be used for data synthesis. If the *I*^2^ test is between 50% and 75%, the random-effects model will be conducted for data synthesis. If the *I*^2^ test is higher than 75%, we will investigate possible reasons from both clinical and methodological perspectives, and provide a descriptive analysis or conduct subgroup analysis.

If the moxibustion has many different forms, such as direct-moxibustion, herb-partitioned moxibustion, moxa burner moxibustion, or natural moxibustion, they will be classified as 1 group in the meta-analysis. Data will be extracted for all time points if sufficient data are available, and outcomes in different time points will be analyzed. The consistency will be assessed by node-splitting method, which estimates the direct and indirect treatment effect separately. If inconsistency is identified, potential sources of the inconsistency need to be detected.

#### 2.5.5 Sensitivity analysis

To test the robustness of the primary decisions of the review process, sensitivity analysis will be conducted. The principal decision nodes conclude methodological quality, sample size, and the effect of missing data. The meta-analysis will be repeated and studies of lower quality will be excluded. The result will be compared and discussed according to the results.

#### 2.5.6 Grading the quality of evidence

The quality of evidence for all outcomes will be judged using the grading of recommendations assessment, development, and evaluation working group methodology.^[22]^ In this approach, direct evidence from RCTs starts at high quality and can be downgraded based on the risk of bias, indirectness, imprecision, inconsistency (or heterogeneity), and/or publication bias to levels of moderate, low, and very low quality.

## 3 Discussion

PDM is a major public health problem in the world, which seriously affects the quality of life and work efficiency of women, causing great economic losses. Studies have signified that moxibustion is effective for relieving the symptoms of PDM. Nevertheless, currently, no systematic review related to moxibustion for PDM has been published in English. The evaluation of this systematic review will be divided into 4 sections: identification, study inclusion, data extraction, and data synthesis. We hope that this review will give more convincing proof to assist clinicians during the decision-making process when dealing with PDM. This review has some potential limitations. Different types of moxibustion therapies and the efficacy evaluation criteria of urticaria in included trials may cause significant heterogeneity.

## Author contributions

**Conceptualization:** Hao Wang, Xin Hui.

**Data curation:** Hao Wang, Xin Hui.

**Funding acquisition:** Baixiao Zhao.

**Investigation:** Lue Ha, Baixiao Zhao, Qin Yao.

**Methodology:** Hao Wang, Xin Hui.

**Resources:** Baixiao Zhao.

**Software:** Hao Wang, Xin Hui.

**Supervision:** Lue Ha, Baixiao Zhao, Qin Yao.

**Validation:** Lue Ha, Baixiao Zhao, Qin Yao.

**Writing – original draft:** Hao Wang, Xin Hui.

**Writing – review and editing:** Hao Wang, Xin Hui.

Hao Wang orcid: 0000-0002-4637-6527.
